# A knowledge-driven protocol for prediction of proteins of interest with an emphasis on biosynthetic pathways

**DOI:** 10.1016/j.mex.2020.101053

**Published:** 2020-09-02

**Authors:** Adwait G. Joshi, K. Harini, Iyer Meenakshi, K. Mohamed Shafi, Shaik Naseer Pasha, Jarjapu Mahita, Radha Sivarajan Sajeevan, Snehal D. Karpe, Pritha Ghosh, Sathyanarayanan Nitish, A. Gandhimathi, Oommen K. Mathew, Subramanian Hari Prasanna, Manoharan Malini, Eshita Mutt, Mahantesha Naika, Nithin Ravooru, Rajas M. Rao, Prashant N. Shingate, Anshul Sukhwal, Margaret S. Sunitha, Atul K. Upadhyay, Rithvik S. Vinekar, Ramanathan Sowdhamini

**Affiliations:** a National Centre for Biological Sciences (NCBS-TIFR), GKVK campus, Bellary road, Bangalor 560065, Karnataka, India; b The University of Trans-Disciplinary Health Sciences and Technology (TDU), Yelahanka, Bangalore 560064, Karnataka, India; c Department of Biotechnology, Thapar Institute of Engineering and Technology, Patiala 147004, Punjab, India

**Keywords:** Pathway, Homology, Multiple sequence alignment, Functionally important residue, Phylogenetic analysis, FIR, Functionally Important Residue, MSA, Multiple Sequence Alignment, PIM, Percentage Identity Matrix, CHI, Chalcone Flavanone Isomerase, DEPC, Diethyl Pyrocarbonate, GAPDH, Glyceraldehyde-3-phosphate dehydrogenase gene

## Abstract

This protocol describes a stepwise process to identify proteins of interest from a query proteome derived from NGS data. We implemented this protocol on *Moringa oleifera* transcriptome to identify proteins involved in secondary metabolite and vitamin biosynthesis and ion transport. This knowledge-driven protocol identifies proteins using an integrated approach involving sensitive sequence search and evolutionary relationships. We make use of functionally important residues (FIR) specific for the query protein family identified through its homologous sequences and literature. We screen protein hits based on the clustering with true homologues through phylogenetic tree reconstruction complemented with the FIR mapping. The protocol was validated for the protein hits through qRT-PCR and transcriptome quantification. Our protocol demonstrated a higher specificity as compared to other methods, particularly in distinguishing cross-family hits. This protocol was effective in transcriptome data analysis of *M. oleifera* as described in Pasha et al.

• Knowledge-driven protocol to identify secondary metabolite synthesizing protein in a highly specific manner.
• Use of functionally important residues for screening of true hits.
• Beneficial for metabolite pathway reconstruction in any (species, metagenomics) NGS data.

## Graphical abstract

**Figure:**
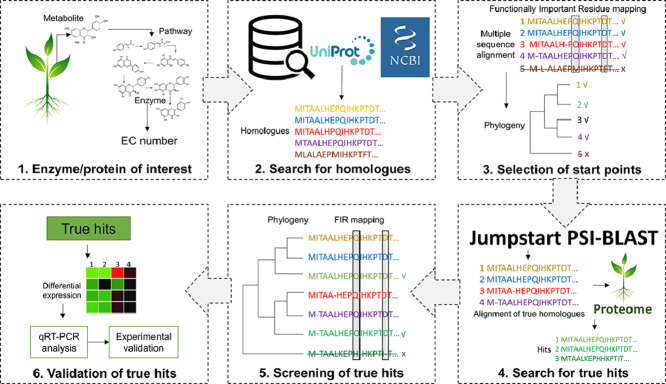

Specification Table

**Table:** 

Subject Area:	Agricultural and Biological Sciences
More specific subject area:	Computational biology, plant genomics
Method name:	Enzyme mining protocol (CAPS_protocol)
Name and reference of original method:	Naseer Pasha S, Shafi KM, Joshi AG, Meenakshi I, Harini K, Mahita J, Sajeevan RS, Karpe SD, Ghosh P, Nitish S, Gandhimathi A, Mathew OK, Hari Prasanna S, Malini M, Mutt E, Naika M, Ravooru N, Rao RM, Shingate PN, Sukhwal A, Sunitha MS, Upadhyay AK, Vinekar RS and Sowdhamini R. The transcriptome enables the identification of candidate genes behind medicinal value of Drumstick tree (*Moringa oleifera*). Genomics, 10.1016/j.ygeno.2019.04.014
Resource availability:	http://caps.ncbs.res.in/download/ddat_methodsx/

## Method

In this paper, we describe an integrative approach to identify enzymes involved in the biosynthesis of secondary metabolites, using closely related homologues for sequence searches, integrated with evolutionary analysis and validation. The method was employed for the identification of candidate proteins involved in the biosynthesis of several secondary metabolites, vitamins and mineral transporters in the transcriptome of *M. oleifera* [1]. The steps involved in this unique pipeline have been described below (Please see Graphical abstract).

1. **Enzymes or protein of interest:** The PlantCyc [2] database was referred to identify the enzymes participating in the biosynthesis of select secondary metabolites, vitamins and mineral ion transporters (listed in Table 1). In case, the pathway information was not available in PlantCyc, the name and EC numbers of the enzymes involved in the metabolite biosynthesis were derived from the literature.

**Table 1:** The counts of sequence hits for enzymes/proteins involved in synthesis of secondary metabolites and vitamins, and metal ion transporters. Set of enzymes in a pathway are numbered according to the biosynthesis reaction steps. The number of hits obtained through the method described here refers to ‘CAPS_protocol’. Comparison with other methods (KAAS, WebMGA, MERCATOR) contains hits matching CAPS_protocol and the number of extra hits identified by individual method is mentioned in brackets.

Pathway/Transporter: Enzyme/Protein	Sequence hits in *M. oleifera* proteome [Hits matching with CAPS_protocol (Extra hits)]			
	CAPS_protocol	KAAS	WebMGA	MERCATOR
**Quercetin: Q1-Q6**				
**Q1**: Coumaroyl CoA ligase	1	1(5)	0(0)	1(3)
**Q2**: Chalcone synthase	2	2(0)	0(0)	2(3)
**Q3**: Chalcone flavanone isomerase	1	1(1)	0(0)	1(2)
**Q4**: Flavanol synthase/Flavanone 3-hydroxylase	1	1(1)	0(0)	1(3)
**Q5**: Tricin synthase	2	2(2)	2(2)	2(0)
**Q6**: Flavonoid 3′-monooxygenase	2	1(0)	2(0)	2(0)
**Benzylamine** (N-substituted formamide deformylase)**:**	1	0(0)	0(0)	0(0)
**Ursolic/Oleanolic acid**				
Amyrin monooxygenase	2	2(2)	2(0)	2(0)
**Vitamin A precursor (Beta carotene)**				
Lycopene beta cyclase	1	1(0)	0(0)	1(3)
**Vitamin C (L-ascorbate): Enzyme1-Enzyme5**				
**Enzyme1**: GDP-D-mannose 3′,5′-epimerase	2	1(0)	0(0)	2(0)
**Enzyme2**: GDP-L-galactose guanyltransferase	2	2(1)	0(0)	2(2)
**Enzyme3**: L-Galactose-1 phosphate phosphatase	1	1(0)	1(3)	1(2)
**Enzyme4**: L-galactose dehydrogenase	1	1(0)	0(0)	1(0)
**Enzyme5**: L-galactono-1,4-lactone dehydrogenase	1	1(0)	1(2)	1(0)
**Vitamin E (alpha-tocopherol)**				
**Enzyme1**: Tocopherol cyclase	1	1(1)	0(0)	1(1)
**Enzyme2**: Gamma tocopherol methyltransferase	1	1(0)	1(7)	1(0)
**Zinc transporter**	3	3(5)	3(5)	3(8)
**Magnesium transporter**	6	4(3)	5(4)	5(7)
**Calcium transporter**	3	3(0)	3(1)	3(0)
**Iron transporter**	3	3(1)	0(0)	3(1)
2. **Search for homologues:** In this step, annotated sequences corresponding to the enzymes in biosynthesis pathway were accumulated. A text-based search was used to identify sequences from protein databases such as UniProt, Non-redundant protein database using the enzyme name or EC number as queries. In case the protein family is poorly represented in UniProt (<20 sequences), sequence homologues of these sequences were identified through BLAST [3] (blastp: https://blast.ncbi.nlm.nih.gov/Blast.cgi). The hits obtained from both the databases were pooled together. The hits were further filtered by employing a query coverage and sequence identity threshold of 70% and 40%, respectively.
3. **Selection of start points:** The screened hits were aligned using Clustal Omega (default parameters, https://www.ebi.ac.uk/Tools/msa/clustalo/) [4] The Functionally Important Residues (FIR) for the selected protein were obtained from the literature and were mapped onto the alignment. FIRs include residues important for catalytic activity, substrate-binding and family-specific residues. Such residues help to distinguish the hits from closely related families and identify the appropriate start points. A phylogenetic tree was constructed using maximum likelihood method from MEGA tool (v7; https://www.megasoftware.net/) [5]. A set of proteins was shortlisted based on the clustering and presence of the FIRs. The sequences, which were lacking the correct FIRs in the alignment, were excluded. Subsequently, the selected protein sequences were considered as the starting point for sequence search in the transcriptome.
4. **Search for true hits:** In this step, we identify the potential candidates from the proteome of *M. oleifera* derived from transcriptome [1]. A multiple sequence alignment (MSA) of the above shortlisted protein sequences was carried out using Clustal Omega [4]. A custom Perl script was used to identify the representative sequence from the MSA. This script identifies that sequence which retains the maximum sequence identity with most of the sequences using the percentage identity matrix (PIM) output file generated by Clustal Omega. The MSA and the representative sequence information were then used as query in a sequence search approach, termed Jumpstart PSI-BLAST [6]. It is a special case of BLAST search, where an alignment can be used to start the sequence search in an iterative manner. During this search, we used expectation value (E-value) of 10^−5^ and an inclusion threshold (*h*-value) of 10^−5^ for two iterations, to identify homologous sequences in the *M. oleifera* proteome derived from the transcriptome [1]. All the hits with >70% query coverage and >40% sequence identity to the representative sequence were taken as potential hits in the *M. oleifera* proteome.
5. **Screening of true hits:** This step enables us to filter the true hits based on the FIR data recorded for the start points. FIRs were mapped onto the alignment (Clustal Omega, default parameters) of hits with the start points to filter the true positives. Additionally, a phylogeny was built using the above MSA derived from start point protein sequences and the putative hits to enable the selection of true (correctly annotated) members of the corresponding enzyme using MEGA v7 [5]. Molecular phylogeny analysis was performed using the Maximum Likelihood (ML) method with 1000 bootstrap iterations to identify the true members of the enzyme family under consideration. The identified hits, which co-clustered in the same clade as the selected start points, also retained conserved FIRs, were selected and the rest of the hits were discarded. This step ensured screening of sequences from the proteome with sequence and functional conservation. In case of enzymes belonging to Cytochrome P450 family (Cyt P450) (Flavonoid monooxygenase: Quercetin pathway, Amyrin monooxygenase: Oleanolic acid/Ursolic acid pathway), some of the sub-families are closely related and have identical FIRs. A curated dataset of Cytochrome P450 sequences from *A. thaliana* from Nelson and coworkers*,* was used to select true hits [7]. A phylogenetic tree was constructed using sequences from this curated database and the identified hits from *M. oleifera* proteome. True positives were selected based on the co-clustering with specific sub-family sequences according to the query enzyme and avoid cross-family hits [8].
6. **Validation of true hits:** The enzymes involved in metabolic pathways and ion transporters, identified through the above steps, were validated using differential gene expression analysis and qRT-PCR. These validation steps are not a part of the current CAPS_protocol, while they have been described in Pasha et al. [1].
  A. Differential expression analysis for the true hits: The *M. oleifera* transcriptome data, as described in Pasha et al. [1], was used for the differential expression analysis. The data was available for five different tissues (flower, leaf, seed, stem and root). The relative abundance of the transcripts across five different tissues was recorded. This enabled tissue-specific association of the true hits. A correlation between tissue-based quantification of metabolites and the relative abundance of their synthesizing transcripts was observed. This supported the identification of the metabolic pathway proteins and transporters identified through our method.
  B. qRT-PCR analysis of true hits: Quantitative RT-PCR provides additional validation for the transcript abundance. We carried out qRT-PCR for transcripts of selected enzymes and ion transporters (as described in Pasha et al.) [1]. A similar trend was observed for tissue-specific abundance as seen in the RNAseq data for most of the transcripts. The qRT-PCR based validation corroborated the identification of proteins through our method.

## Comparison with other methods

The protocol described here (CAPS_protocol) takes into account the currently known protein sequences for an enzyme in a metabolic pathway. Since it was ensured that the start points are annotated enzymes, it served as a knowledge-based sequence identification for metabolic pathways in the query proteome/genome/transcriptome. We compared this strategy with some of the freely available and accessible webservers for metabolic pathway enzyme identification. We compared results from ‘KAAS’ (https://www.genome.jp/kegg/kaas/) [9], WebMGA (http://weizhong-lab.ucsd.edu/webMGA/) [10] and MERCATOR (https://mapman.gabipd.org/app/mercator) [11] with CAPS_protocol (Table 1, Supplementary Table S1). The KAAS webserver renders functional annotation through BLAST comparisons with the KEGG GENES database and the subsequent KEGG Orthology assignments enables pathway mapping [9]. The WebMGA server provides the KOG entry for the query sequence which has to be subsequently associated with the protein's function [10]. The MERCATOR web server implements combination of BLAST-based and protein domain-based searches to compute the bin assignments used in their back-end algorithm and data library. Since, these are based on the annotated data from several plant species, it provides direct relation for the plant-based queries [11].

The entire protein set was used as a query for all three webservers. We combined the results for identification of proteins involved in ion/mineral transport or metabolic pathways, as discussed in Pasha et al. [1]. Table 1 lists the proteins identified in the *M. oleifera* transcriptome through CAPS_protocol and through the webservers (KAAS, WebMGA and MERCATOR). There were 37 transcriptome-derived proteins sequences observed for 20 proteins (multiple hits) spread across 10 different metabolic pathway enzymes and metal ion transporters (Table 1). Among the three compared webservers, MERCATOR performed best and could identify 36 out of 37 of gene products (97%), clearly indicating the advantage of using plant-based resources for annotation. KAAS identified 33 (89%) and WebMGA identified 17 (45%) gene products. All three webservers identified several extra hits for many of the proteins that were missed or filtered by CAPS_protocol during functionally important residue (FIR) mapping and clustering. There was an over representation of some enzymes with a generic annotation (example: methyltransferase, cytochrome P450) as potential hits (as described in Step 3 of Methods section). On the contrary, CAPS_protocol could render annotation to the exact enzyme in the metabolic pathway for the gene products. Therefore, CAPS_protocol was more specific towards the metabolic pathway annotation in the proteome, as compared to other webservers considered here. The knowledge-based sequence search initiated for functional annotation, assisted with the FIR mapping and subsequent clustering, thus provides advantage for identification of gene products in the transcriptome.

## A case study to identify chalcone flavanone isomerase (CHI) enzyme from *M. oleifera* proteome

**Fig. 1:**
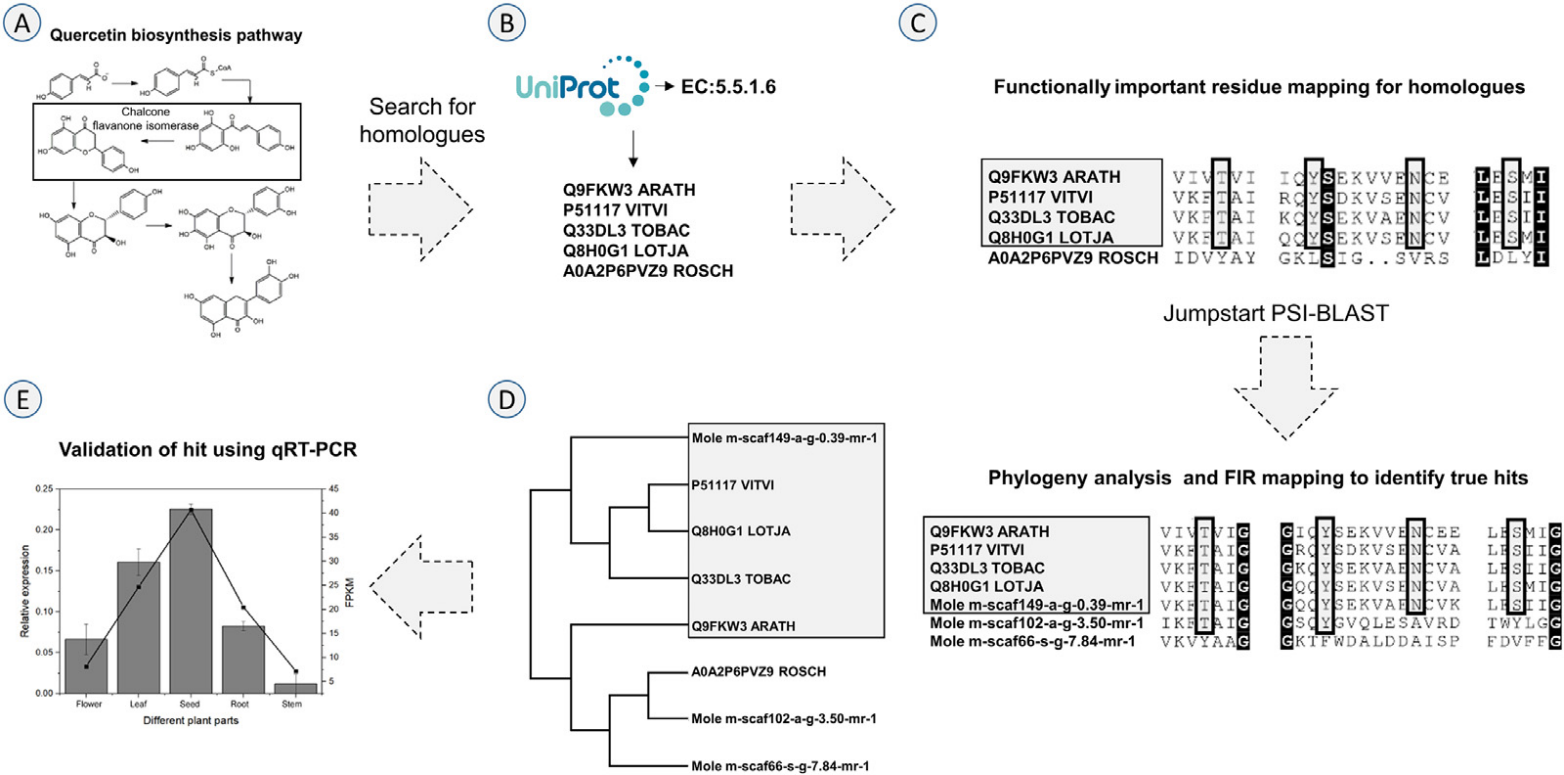
A case study to demonstrate the enzyme mining protocol. A) Quercetin biosynthesis pathway (chalconeflavanone isomerase (CHI)). B) Homologue search using EC number of enzyme in UniProt database. C) Functional ImportantResidue mapping (residue block) to identify true hits (grey colour) from the alignment of homologues and the alignment usedfor Jumpstart PSI-BLAST search. D) FIR mapping (residue block) and phylogenetic analysis performed to identify true enzymehits (grey colour) from our proteome. E) The identified hit were further validated using tissue-based quantification by qRT-PCR(bars) and transcriptome analysis (line).

Fig. 1. A case study to demonstrate the enzyme mining protocol. (A) Quercetin biosynthesis pathway (chalcone flavanone isomerase (CHI)). (B) Homologue search using EC number of enzyme in UniProt database. (C) Functional Important Residue mapping (residue block) to identify true hits (grey colour) from the alignment of homologues and the alignment used for Jumpstart PSI-BLAST search. (D) FIR mapping (residue block) and phylogenetic analysis performed to identify true enzyme hits (grey colour) from our proteome. (E) The identified hit were further validated using tissue-based quantification by qRT-PCR (bars) and transcriptome analysis (line).

As a case study, we selected one of the enzymes from quercetin biosynthesis pathway (chalcone flavanone isomerase) for the enzyme mining from *M. oleifera* proteome using CAPS_protocol (Fig. 1: (A)). We collected the pathway information from PlantCyc database [2]. The enzyme name chalcone flavanone isomerase (CHI) and information (EC: 5.5.1.6) were used as keywords to perform a text-based search against UniProt database to identify annotated homologues in other plant species (Fig. 1: (B)) [12]. All the homologous sequences were further aligned using Clustal Omega with default parameters [4]. The FIRs collected from the literature and UniProt (Q9FKW3_ARATH), such as binding site residues like Threonine (T41), Asparagine (N106) and Serine (S183) and a catalytic residue Tyrosine (Y99), were mapped to the alignment to recognise true homologues (Fig. 1: (C)) [12]. We further removed the sequences that were not matching FIRs in the alignment. The alignment of these true homologues was used as a query to perform Jumpstart PSI-BLAST against our *M. oleifera* proteome by employing an E-value threshold of 10^−5^ to identify enzyme hits as described above in the method (Step 4) (Fig. 1: (D)). Further, the hits were filtered by giving percentage identity and query coverage threshold, above 40% and 70%, respectively. The alignment with the proteome-wide hits was further analysed for FIR mapping. We then constructed a maximum likelihood phylogenetic tree, using MEGA, with 1000 bootstrap iterations to infer the clustering [5]. We also considered the hits from KAAS, WebMGA and MERCATOR for comparison with our predicted hits. The predicted gene product ‘Mole_m-scaf149-a-g-0.39-mr-1’ was considered as true hit, since we could map all the FIRs and it clustered with the other known homologues. The hits from other tools ‘Mole_m-scaf102-a-g-3.50-mr-1’ and ‘Mole_m-scaf66-s-g-7.84-mr-1’ failed to show the FIRs and did not cluster with the known true homologues. Additional validation has been done (as described in Method step 6: A, B) for the true hits using qRT-PCR (Fig. 1: (E) – bars) and correlated the expression (Fig. 1: (E) – line) for each sample from the transcriptome analysis. This corroborated the tissue-wide differential expression with qRT-PCR quantification for CHI. Thus, we have demonstrated that CAPS_protocol can be used to identify the secondary metabolite synthesizing enzymes and other proteins of interest from a given query proteome (obtained from transcriptome or genome).

## Limitations of CAPS_protocol

The CAPS_protocol is predominantly a knowledge-driven approach for the identification of potential enzymes involved in the synthesis of secondary metabolites in NGS data. Despite its utility, there are certain limitations to this approach as described below:

*Necessity of manual inspection*: This approach relies on manual curation of start points, FIRs, etc. before initiating the sequence mining in the NGS data. This ensures high fidelity for identifying exact matches for the protein of interest. However, this can be potentially observed as a limitation, since, these steps cannot be automated and have to be addressed on a case-by-case basis.

*Lack of package or webserver*: This protocol requires manual inspection in few steps. This makes it difficult to provide it as a package or host it on web-server/web-services, unlike the other methods compared above (like KAAS, MERCATOR and WEBMGA). The lack of a web-server is a potential limitation for the end-user.

*Automated large-scale analysis*: The NGS data analysis, typically processes large-scale data in an automated fashion. However, due to lack of automation, CAPS_protocol has a limitation to process large number of cases in a single run. Therefore, it has to be utilized in a batch process, where each case is dealt with using the necessary manual curation. Hence, compared to other methods (discussed above), this method will take more time.

## Additional information

### Background

The synthesis of particular secondary metabolites involves several biochemical reactions, starting from a substrate, going through several intermediates and finally culminating in the formation of the product. Enzymes involved in each biochemical reaction step can be selected from annotated metabolite pathways in various databases and literature. However, establishing a direct association between the known proteins in the newly identified genome/transcriptome is challenging. Several tools are available for identification of metabolic pathway enzymes; however, they often tend to identify non-specific proteins. Additional validation steps, as described above, in this method ensure selection of true hits based on sequence and functional conservation.

## Additional information on validation methods

### Plant material harvesting and Total RNA isolation

The *M. oleifera* plant tissues (Flower, leaf, seed, root and stem) were obtained from the University of Agricultural Sciences, GKVK, Bangalore, India. The freshly collected samples were rinsed with diethyl pyrocarbonate (DEPC) treated RNase free water and blotted on autoclaved filter paper. Aluminium foil was used for covering the samples and flash frozen in liquid nitrogen and stored at -80°C until used. All the five tissues were collected from three different individuals that forms the biological replication. All the protective precautions and extreme care were taken while collecting the samples to minimize the RNase contamination. All the materials used were treated with DEPC treated water, autoclaved and dried in hot air oven prior to RNA isolation. Total RNA from the different tissues were isolated using Spectrum Plant total RNA kit (Sigma Aldrich) as per manufacturer's guidelines. The quality and quantity of the extracted total RNA was analysed by denaturing formaldehyde agarose gel electrophoresis and BioSpec-nano Micro-volume UV-Vis Spectrophotometer (Shimadzu, USA), respectively [1].

## First strand cDNA synthesis and qRT-PCR protocol

The protocol for qRT-PCR, used to validate the true hits for the enzymes in the metabolic pathways has been described in the supplementary data (Additional methods and results) of Pasha et al*.* [1].

## Declaration of Competing Interest

The authors declare that they have no known competing financial interests or personal relationships that could have appeared to influence the work reported in this paper.

## Appendix Supplementary materials

**Supplementary Table (S1)**: The identifiers of sequence hits for enzymes/proteins involved in synthesis of secondary metabolites and vitamins, and metal ion transporters. Set of enzymes in a pathway are numbered according to the biosynthesis reaction steps. Hits, obtained through the method described here, are provided under column CAPS_protocol. The sequence identifiers of hits obtained through additional methods (KAAS, WebMGA, MERCATOR) are also listed in respective columns. Image, application 1
